# Observations on Chronic Diarrhæa, Arising from an Ulcerated State of the Intestines

**Published:** 1814-11

**Authors:** John Gorham


*For the Medical and Physical Journal.*

### *Observations on Chronic Diarrhœa, arising from an Ulcerated State of the Intestines;* by JOHN GORHAM, M.D.

THE subjects of this disease are found in the lower classes of society, and it is most frequently met with in hospital practice. Having had several opportunities of witnessing its progress, and, after death, of ascertaining by dissection the morbid changes in the intestines, on which it depended, I have thought that a concise account of its symptoms, and of the influence of medicine in relieving them, might, perhaps, afford some useful hints to those who are entrusted with the medical department of such public institutions.

The disease is generally confirmed before the patient is admitted; and the physician can do little more than record protracted sufferings and a fatal termination. The most prominent symptom is a purging, more or less severe: the stools are thin and frothy, sometimes slimy, small in quantity, and occasionally mixed with blood, and rarely with pus. The discharge is preceded by pain about the umbilicus, and frequently succeeded by violent straining and tenesmus. The number of discharges varies exceedingly in different cases, but in all it is increased at night. The pulse is in some moderately full and frequent, in others small and quick; the skin is dry and husky; the tongue either dry, parched, and covered with a thin whitish crust, or clean and moist; a clammy or bitterish taste is frequently perceived; the thirst is always urgent; the appetite for the most part is much impaired, but in a few cases it has been good until within a few days of death. With these symptoms are usually joined those of dyspepsia, such as eructations, a sense of fulness and of weight about the præcordia, heartburn, and occasional pain and vomiting after eating.

As the disease advances, the countenance becomes sallow and sharp, the fat in the cellular membrane is gradually absorbed, and the body exhibits great emaciation; the patient complains of considerable debility; slight rigors are experienced, rienced, followed by flushings of the face; pains are felt about the neck and shoulders, and a stiffness is perceived in the lower extremities; serous effusions take place first in the feet, and the œdema extends along the legs even to the penis and scrotum. These are followed by ascites and hydrothorax, and in one case I have seen the effusion even in the face and upper extremities. At this period the urine is scanty, high-coloured, and deposits on standing a flocculent mucous substance, or a greyish white, sometimes a reddish sediment in considerable quantities. The lips lose their colour, the countenance is meagre and pale, or saturnine and of a waxy appearance; difficulty of respiration harasses the patient; the debility becomes extreme; and a miserable existence is at length terminated from the effects of pain, inanition, and universal dropsy.

These symptoms vary greatly in degree in different cases. In all, the purging is the most obvious and most constant; but in some, the appearances of effusion into the cavities of the abdomen and thorax are wanting; and in others, the quantity of blood discharged is small, and there have been no indications of pus.

On repeated dissections, the following morbid changes have been observed.

The muscles were thin: they had nearly lost their red colour, and had the appearance of having been macerated. Serous fluid issued from the cellular texture. The abdomen contained a yellowish translucid fluid, varying in quantity in different cases. The liver was generally shrunk, the gall-bladder distended, although the ducts were pervious, and the spleen and mesenteric glands were enlarged. The stomach, duodenum, jejunum, and part of the ileum, were often found without any peculiar marks of disease; but in one or two instances, all these portions of the alimentary canal exhibited erosions of the internal coat. For the most part, the seat of the disease has been in the lower portion of the ileum, in the cœcum and the colon almost to the rectum. When these parts were examined, a number of erosions or ulcerations were discovered, varying in extent from one line to more than an inch in diameter, the villous coat in all having been destroyed, and in some even the muscular fibres of the middle coat. I have generally found these ulcers to be most numerous at or near the junctions of the ileum and colon with the cœcum. The number, however, varied in different cases. In some, there were but a few of a large size, while in others they were exceedingly multiplied. The edges of these ulcers were slightly elevated, jagged, and irregular, and and a diffused redness appeared in the peritoneal coat immediately over them. Their surfaces were usually covered with a tenacious mucus, and sometimes, though rarely, the intestine around and the peritoneal covering were sphacelated. The engraving of these ulcers, given in the works of Dr. Stark, is very accurate.* From the long continued purging in this disease, it might be supposed that the intestines would contain nothing but flatus, but, in several cases, the cœcum and part of the colon were found filled, though not distended, with dark-coloured tenacious fæces of an intolerable odour. In two cases, lumbrici were discovered in the ilium.

The origin of this disease can generally be traced to exposure to cold. The subjects of it are those who are addicted to the immoderate use of ardent spirits, and who, while intoxicated, have been exposed to the vicissitudes of the seasons, or to the night air, without any other protection from the weather than their clothes. It may, however, be produced by other causes more immediately applied to the internal surfaces of the intestines. Highly acrid substances, or drastic purgatives taken into the stomach, may bring on hyper-catharsis and inflammation, which shall terminate in ulceration; and I have now under my care a deplorable case of this disease, the consequence of confiding in the promises, and taking the medicines, of a notorious empyric.

This complaint is of a chronic nature. Its progress is slow, and it gradually wastes the body of the patient by almost imperceptible degrees. Its usual period, particularly when the most prominent symptoms have been in some measure alleviated by appropriate medicine, is from one to four years. It is commonly, if not uniformly, fatal; and this unfortunate termination is to be accounted for, not only from the nature and seat of the disease itself, but also from its being frequently complicated with organic affections of the other abdominal viscera.

From its seat, it is obvious that the diseased part must be continually irritated by the application of substances taken into the stomach as food, but which, from the imperfect state in which the functions of that organ are performed, are not completely digested. By their stimulant operation, they increase the natural motion of the muscular fibres: this action is probably communicated to the exhalents and mucous follicles, by which a larger quantity of fluid is poured into the

---

* Stark's Works, 4to. London, 1788.

intestines, intestines, and the discharge, if not continually increasing, is at least rendered permanent. From the impossibility of making any direct application to the ulcerated surface, we are prevented from using the only means which could be successful; and when we add to this the previous habits of the patient, and the probability that there exists disease in some or all of the other chylopoietic viscera, we shall not be at a loss to account for its fatality. Such morbid changes in the abdominal organs, independent of those in the intestines, I have frequently observed. In one case, ulcerations about the cœcum were accompanied with extensive disease around the pyloric orifice of the stomach; in a second, with a singular change of structure in the liver, the substance of which appeared to have burst its investing membrane in a great number of places, and to have been protruded, resembling a liver which has been long boiled; in a third, with an abscess between the rectum and the posterior portion of the uterus, which was ulcerated and laid open. The appendix cœci adhered closely to the walls of the abscess, and had allowed the passage of a lumbricus from the intestine to the uterus, in the cavity of which it was coiled; for this appendix penetrated into the abscess: when examined, it was found open at both ends, and its bore corresponded with the diameter of the worm. In a fourth case, which was that of a maniac, this disease was complicated with inflammation of the intestines and peritoneum, with large biliary concretions, and with a sphacelus of one portion of the ilium, through which a lumbricus had penetrated into the cavity of the abdomen. With such alterations in structure, and such obvious derangement of functions, as were indicated by the symptoms, and exhibited in the examinations, of these cases, it is apparent that no mitigation, and much less a cure, could be expected from medicine.

As this disease of the intestines arises from an inflammation of their internal coat, it is probable, that, if properly treated at its commencement, it might in most cases be speedily removed. But after it has afflicted the patient for weeks, or months, it can no longer be regarded as enteritis; and it will be found by experience, that, in general, the only indication to be kept in view is to palliate the symptoms, and render the condition of the patient less miserable.

I shall briefly mention the medicines which have been administered for the purpose, either of effecting a cure, or of restraining the discharge, and the result of their action.

*Opium.*—When this narcotic is given alone, it at first relieves the pain and moderates the discharge in a very considerable degree, and the patient is enlivened with the pleasing idea that his health will soon be restored. The same effect is produced by injections of mucilaginous liquids mixed with the tincture of opium. The influence of this medicine on the diarrhœa, however, is rarely perceptible beyond a week or ten days, and to produce any degree of alleviation, the dose requires to be rapidly augmented. A habit of taking this drug is thus soon established. The person, while the system is excited by it, is rendered comparatively comfortable; but, during the intervals, the debility is great, and existence becomes a burthen. The only benefit to be derived from its use, is that of relieving pain, and procuring a few hours of unrefreshing sleep.

*Ipecacuanha and Antimony.*—In those cases where the pulse has been fu l and frequent, the skin hot and dry, and the strength not much reduced, an emetic of ipecacuanha, followed by small and frequently repeated doses of the same root, has had a beneficial effect by determining to the skin, lessening the quantity of fluids poured into the intestines, and weakening the peristaltic motion.

Of the preparations of antimony, the vitrified oxide with wax, recommended by Dr. Stark, has produced the best effect. In was given in a dose of five grains, combined with one of opium, every sixth hour. In one case, it suspended the discharge and rendered the bowels regular for nearly two months. The patient began to recover his strength and appetite, and I anticipated a cure, but the flux returned, and the man died. This case, as I ascertained by dissection, was an ulcerated state of the intestines, apparently unconnected with disease of any other of the abdominal viscera, notwithstanding large serous effusions had taken place in the cavities and the cellular texture.

I have seen a great abatement of all the symptoms follow the use of the warm bath and subsequent frictions. By this mode, and by the use of opium and astringent medicines, one patient was so much relieved, that could the former have been continued, which, from certain circumstances, was quite impracticable, it is probable that a cure might have been effected. This method is best adapted to the disease while recent.

*Purgatives*—So far as my experience extends, the use of this class of medicines is injurious. When the dyspeptic symptoms have been troublesome, a temporary benefit has been derived from the exhibition of rhubarb in powder, or in tincture. The latter, perhaps, is preferable.

*Tonics and Astringents.*—Cinchona and the bitters have had little influence in checking the progress of this disease. The former, in the state of powder, is injurious, as it always increases increases the number of discharges, and often augments the pain.

Of all the medicines which I have tried, the astringents appear to have had the greatest effect in restraining the profuse diarrhœa.

Of those from the vegetable kingdom, I have most generally employed Kino and Extract of Catechu, in the form of tincture, that of the former being combined with tincture of opium, in the proportion of an ounce of the one, to a half or a whole drachm of the other.

Among the metallic tonics and astringents, the acetate of lead and the sulphate of zinc, have operated most favourably on this disease. The former should be combined with opium, or with this narcotic and root of ipecacuanha, as in the following formula:

℞. Pulveris, Radicis Ipecacuanhæ, gr. xii.

Acetatis Plumbi.

Pulveris Opii. ana. gr. ix.

Mucilaginis. G. Mimosæ niloticæ, quantum satis sit m. fiat massa, in pillulas duodecim æquales dividenda, quarum unam omni sexta hora, capiat.

The quantity of acetate of lead and of opium may soon be augmented to one grain in each pill, but the use of this metallic salt should not be long continued.

The sulphate of zinc may be conveniently exhibited, dissolved in a decoction of the wood of quassia, or the bark of cascarilla.

*Quicksilver.*—The submuriate, the only preparation of this powerful stimulant which I have exhibited, has never, to my knowledge, produced the least favourable effect on the disease, or the least remission in the severity of the symptoms. It has been given alone, in combination with opium, and with opium and ipecacuanha, until it excited ptyalism, without any benefit being perceived to result from its action.

Blistering over the abdomen has sometimes had a temporary good effect, and perhaps a perpetual blister, a seton, or an issue, might be permanently useful, but neither of these has been tried.

I shall now close these brief observations, with a very few remarks on the diet best calculated for those afflicted with this disease. It should be nourishing, and at the same time composed of those substances which are of easy digestion, and which are not much disposed to become ascescent, such as tender meats, eggs, milk, jellies, and simple soups. Except in protracted cases, the functions of the stomach are rarely so far deranged as to prevent the digestion of animal food; and it is only in the advanced stages that a true

 lientery tientery comes on, and the food can be perceived to constitute a part of the alvine dejections. The drink should consist of whey and diluted wine or brandy. Vegetable food in general, and all fermented liquors, such as cyder, beer, and ale, should be avoided, as they uniformly produce a sense of fulness and distension in the abdomen, and augment the number of discharges.—*New-England Journal.*

